# Graphitic Carbon Nitride as a Sustainable Photocatalyst Material for Pollutants Removal. State-of-the Art, Preliminary Tests and Application Perspectives

**DOI:** 10.3390/ma14237368

**Published:** 2021-12-01

**Authors:** Daniele Cecconet, Michela Sturini, Lorenzo Malavasi, Andrea G. Capodaglio

**Affiliations:** 1 Department of Civil Engineering & Architecture, University of Pavia, 27100 Pavia, Italy; daniele.cecconet@unipv.it; 2 Department of Chemistry, University of Pavia, 27100 Pavia, Italy; michela.sturini@unipv.it (M.S.); lorenzo.malavasi@unipv.it (L.M.)

**Keywords:** photocathalysis, AOPs, graphitic carbon nitride, adsorption, Xenon lamp, titanium dioxide, organic pollutants

## Abstract

Photocatalysis is an attractive strategy for emerging pollutants remediation. Research towards the development of new, efficient and effective catalytic materials with high activity under wide irradiation spectra is a highly active sector in material science. Various semiconductor materials have been employed as photocatalysts, including TiO_2_, SrTiO_3_, CdS, BiVO_4_, Ta_3_N_5_, TaON, Ag_3_PO_4_, and g-C_3_N_4_. The latter is a metal-free, low cost polymer, providing high adsorption and catalytic properties, shown to be promising for photocatalysis applications under visible light. Furthermore, g-C_3_N_4_ composites are among the most promising advanced photocatalytical materials that can be produced by green synthesis processes. In this paper, the state-of-the-art of g-C_3_N_4_ applications is reviewed, and application perspectives are discussed. Photocatalysis tests with g-C_3_N_4_ under Xenon irradiation were performed to gather first-hand information to improve photoreactor design. Xenon light spectrum appears to be a suitable radiation source to replace direct sunlight in engineered pollutants removal processes catalyzed by g-C_3_N_4_, in lieu of other currently used heterogeneous photocatalysis processes (e.g., TiO_2_-UV). LED sources are also very promising due to higher energy efficiency and customizable, catalyzer-specific irradiation spectra.

## 1. Introduction

The search for energy-efficient pollutant degradation strategies has a prominent role to sustainably address many current environmental problems, especially relating to the elimination of emerging contaminants. Water and wastewater treatment processes are in fact quite energy and emission intensive [1]. Photocatalysis has been the focus of considerable attention in recent years, and has been applied in a variety of processes across a broad range of industrial fields, including environmental pollutants remediation. First observed as the “Honda–Fujishima effect”, which consists of photo-electrochemical water splitting in the presence of TiO_2_, heterogeneous photocatalysis has originated an important class of processes for environmental remediation, disinfection, energy production, and synthesis of organic compounds. The involved reactions are induced by a solid catalyst that uses photonic energy as the driving force to induce chemical transformations. The process, often proposed for the removal of persistent contaminants that cannot easily be degraded by conventional means, is usually classified as an Advanced Oxidation Process (AOP), a class of processes based on the production of strong oxidative radicals capable of degrading organic molecules in solution. However, as photocatalysis is not only able to oxidize, but also to reduce dissolved components, it should actually be considered an Advanced Oxidation/Reduction Process (AORP) [2].

Photocatalytic processes develop through electronically excited states induced in a catalyst (usually a semiconductor material) through light (energy) absorption, and is normally associated with a series of adsorption/desorption and surface reactions, with overall kinetics governed by the slowest step. A catalyst is characterized by a conduction band (CB) and a valence band (VB), separated by an energy band gap (E_G_). The redox reactions occur when the semiconducting catalyst is excited by photons with energy equal or higher than its band gap energy, so that electrons receiving that energy will jump from VB to CB [3].

Photocatalytic reactions, i.e., oxidation of electron donors and reduction of acceptors, respectively, occur at the interface between semiconductor and liquid medium. Electrons and holes migrate to the surface of the semiconductor and can reduce and/or oxidize, respectively, reactants adsorbed on the surface, depending on the substrate’s redox potential. An adsorbed molecule is reduced if its reduction potential is higher than that of the photoelectrons or it can be oxidized if its potential is lower than that of the photohole. As in most AORP reactions, active species such as hydroxyl radicals (•OH), superoxides (O_2_•, HOO•) and holes, are formed in the solution by the incoming energy [4]. Hydroxyl radicals are the main oxidants in aqueous solutions, generated by direct hole oxidation or photogenerated electron-induced O_2_ reduction, according to the sequence:

(a) O_2_ + e^−^ = O_2_•
(b) O_2_• + e^−^ + 2H^+^ = H_2_O_2_
(c) H_2_O_2_ + e^−^ = •OH + OH^−^

In most cases, photodegradation kinetics at low pollutant concentrations follow the Langmuir–Hinshelwood model, under a first order reaction. Usually, the overall reaction rate of photocatalytic processes is slow compared to other processes’ reaction rates, but this can be overcome in practical applications by providing a greater amount of active catalyst in the reactor.

Photocatalytic activity depends greatly on the wavelength and intensity of incident light and on semiconductor photocatalyst’s intrinsic band structure, which determines its electronic transport properties [5]. Efficient harnessing of this energy remains an issue in process efficiency [6], requiring suitable materials with narrow band gaps, a high quantum yield, an efficient transport of charge carriers, and good stability. On an energy basis, the solar spectrum consists of about 3–5% UV (λ < 400 nm), about 45–47% visible light (400 < λ < 700 nm) and about 47–50% IR (700 < λ < 1000 nm) [7]. In natural conditions, visible light activated photocatalysis could exploit at best the incoming solar energy. Availability of efficient catalysts compatible with natural or artificial light sources is essential for determining the overall success of photocatalysis: in the past few years, various semiconductor materials have been extensively employed as catalysts for different redox reactions. Among these are TiO_2_, SrTiO_3_, CdS, BiVO_4_, Ta_3_N_5_, TaON, Ag_3_PO_4_ and, recently, g-C_3_N_4_.

In particular, titanium dioxide (TiO_2_), an inert and safe material in bulk form, is by far the most widely studied photocatalyst, with many potential applications in the decomposition of organic pollutants due to its excellent oxidizing capabilities [8]. The commercial form Degussa P25 is considered to be the research standard, with well-defined structural characteristics (70% anatase and 30% rutile nonporous crystal structure, with band gap energy of 3.2 eV and 3.0 eV, respectively), a large surface area of 50 m^2^/g, and an average particle size of 30 nm [9]. However, TiO_2_ is activated only by ultraviolet (UV) light (λ < 400 nm) adsorption, which greatly limits its practical application. The need for UV irradiation sources in the most commonly studied photocatalytic processes has limited their actual feasibility and potential environmental benefits at an industrially relevant application scale [10]. Mercury gas discharge lamps, in fact, are a common UV radiation source, but have limited energetic efficiency. Commercial low pressure discharge lamps produce UV light at a wavelength between 180 and 254 nm with energetic efficiency of around 32%, while medium pressure discharge lamps produce UV light on a more ample spectrum from 180 to 400 nm, but with a lower energetic efficiency (around 12%).

In addition, even though it is one of the most studied materials for photocatalytic applications, TiO_2_’s quantum yield (i.e., moles of compounds oxidized over moles of photons absorbed) is lower than 5%. The quantum yield cannot easily be improved, as it is a fundamental property of the material. To improve a catalyst’s light absorption response, the main strategy consists of improving the number of light-active sites on its surface, and restricting the charge–carrier recombination: an enhancement of light-active sites can be obtained by improving the catalyst’ s surface area, and/or increasing the amount of active facets of the material. Charge–carrier separation can be improved by morphology and heterostructure construction modification with a suitable band alignment. The presence of open pores can allow reactants’ diffusion within the photocatalyst material itself [11].

Although TiO_2_ visible light absorption may be improved by impurity doping, sensitization, surface modification, and fabrication of composites, these may introduce drawbacks that could limit actual applications, and require complex additional synthesis steps that may be unpractical or overly costly [12]. Despite the high commercial cost (up to 500 €/kg), the prevalence of nanosized TiO_2_ forms has exponentially increased in photocatalytic studies over the past decade, since they have been shown to be most effective in process applications [13]. However, experimental evidence from animal studies has led to the classification of TiO_2_ nanoparticles as a possible carcinogenic to humans in general, and as occupational carcinogens specifically; furthermore, nano-TiO_2_ has demonstrated several other adverse effects on human health, such as oxidative stress induction, resulting in possible cell damage, genotoxicity, inflammation, immune response, and more [14]. These findings suggest that the use of TiO_2_ nanoparticles in actual applications would require special precautions, such as the limitation of direct human contact, and their removal from treated solutions prior to their release to the environment. All these factors have spurred research towards the development of alternative catalytic materials with high activity under wide spectrum irradiation and a negligible environmental impact for photocatalytic applications.

For these reasons, graphitic carbon nitride (g-C_3_N_4_), a metal-free, low cost semiconductor polymer, has achieved much interest in photocatalysis due to its moderate band gap of 2.7–2.8 eV, corresponding to the 450–460 nm wavelength of the solar spectrum. Its negative conduction band (CB) potential (−1.3 V vs. NHE at pH 7.0) and positive valence band (VB) potential (1.4 V vs. NHE at pH 7.0) allow it to induce photocatalytic redox reactions under natural or simulated solar irradiation. Photocatalytic H_2_ production with g-C_3_N_4_ was observed by Wang et al. in 2009 [15], and its good performance in photooxidation of organic pollutants under visible light irradiation was later confirmed by Yan et al. [16] When synthesized, g-C_3_N_4_ has a flake-like structure similar to that of graphite, with a high internal surface area and unique stability properties, being non-volatile up to 600 °C. Carbon nitride materials have been shown to be among the most promising new metal-free eco-friendly catalyst materials for photocatalytic applications due a number of attractive properties, including wear resistance, chemical and thermal endurance, hardness, low density, water resistivity, biocompatibility, and stability in an ambient environment. g-C3N4-based nanostructures have emerged as ideal catalyst candidates for many environmental (and energy) photocatalysis applications, including pollutant degradation.

This paper analyses reported applications of g-C_3_N_4_-based adsorption and photocatalysis processes for pollutant removal, and discusses the requirements for efficient implementation of engineered carbon nitride-based photocatalysys, based on preliminary tests for removal of Methylene Blue in solution under varying conditions.

## 2. g-C_3_N_4_ Synthesis, Properties and Applications

g-C_3_N_4_ is formed of two abundant and readily available elements, C and N, suggesting that it can be synthesized easily and at low cost. Titanium, by comparison, although the 9th most abundant element on Earth (7th most abundant metal), commands a relatively high price as a result of high extraction costs, energy requirements and material losses (about 90%) involved in the production process of the pure mineral.

Synthesis methods to prepare g-C_3_N_4_, on the other hand, include an easily performed thermal condensation at between 500–600 °C from low cost, nitrogen-rich, easily accessible organic precursors (some of them recoverable as residuals/byproducts of common industrial processes) such as: urea, melamine, thiourea, cyanamide, dicyanamide, or guanidine hydrochloride. These are the building blocks of the basic tectonic units, like s-triazine (C_3_N_3_) and tri-s-triazine (C_6_N_7_) rings, which depend mainly on the synthesis process that involves nucleophilic addition, polycondensation and polymerization [17]. Usually, g-C_3_N_4_ is polymerized after rearranging melamine and dicyandiamide intermediates into tri-s-triazine at a temperature of approximately 390 °C. Once heated to about 520 °C, g-C_3_N_4_ emerges from the basic units; disordered structures may also be synthesized due to incomplete intermediate removal. Crystalline g-C_3_N_4_ can be prepared by various techniques, like ionothermal (molten salt), molecular self-assembly, microwave irradiation, and ionic liquid synthesis [18]. The synthesis is affected by reactor’s atmosphere, which influences the intrinsic structure and properties of resulting g-C_3_N_4_. To optimize the physicochemical properties of the final molecule, precursors may be pretreated prior to thermal processing, for example by protonation or sulfur-mediated synthesis [19].

Compared to TiO_2_, g-C_3_N_4_ shows much better absorption characteristics of the visible light spectrum. An observed internal quantum yield of 26.5% (more than five times that of TiO_2_) under visible light was reported by Martin et al. [20] Light absorption can be further enhanced by metal entrapping to form active sites with an improved photocatalytic performance, which is considered quite important for green chemistry developments [21]. On the other hand, g-C_3_N_4_ may occasionally exhibit low photocatalytic efficiency due to some flaws of the material itself, such as: insufficient visible absorption below 460 nm, a low surface area (∼10 m^2^/g), a high degree of monomer condensation, small active sites for photoreactions, slow surface reaction kinetics, moderate oxidation ability, boundary effects and low charge mobility, and high electron hole recombination rates [22]. Due to its properties, basic g-C_3_N_4_ normally cannot achieve a very high formation of hydroxyl radicals from water oxidation, but only oxygen evolution. This confers the material possibility of achieving various photooxidation transformations of organic solutes, but not a significant direct mineralization by the effect of strong hydroxyl radicals [23]. This limitation can be overcome by particular fabrication techniques.

### g-C3N4 for Pollutant Control

Adsorption is one of the earlier and simpler techniques used for the elimination of pollutants on a large scale, and constitutes the first step of many photocatalytic reactions. The specific properties (abundant active sites, functional groups and high specific surface areas) of g-C_3_N_4_ provide a high capacity and fast kinetics for the adsorption of metal ions or organic pollutants through different kinds of interaction mechanisms. These are dominated by the organic molecules’ structures, the adsorbents’ surface functional groups and structure, and by the solution’s pH, temperature, and composition (e.g., co-presence of different organic/inorganic pollutants). For example, adsorption of methylene blue (MB) on biochar/g-C_3_N_4_ composites showed the removal efficiency of 96.7%, with an overall adsorption capacity of 302.3 mg/g at pH = 11 [19]. Malachite Green, Methyl Orange, and Rhodamine B were adsorbed at pH 6 at 160, 192 and 202 mg/g, respectively on g-C_3_N_4_, with an increased efficiency of up to 201, 221 and 482 mg/g, respectively on a KMnO_4_ oxidized composite [24]. Rhodamine B was adsorbed at pH 4 at a staggering 2900 mg/g on a Fe_3_O_4_/ZIF-8 magnetically recyclable g-C_3_N_4_ nanocomposite [25].

Apart from the specific ionic species and individual composite properties (type of functional groups, charge and surface sites densities), g-C_3_N_4_ sorption of metal ions strongly depends on process conditions, particularly solution pH, ionic strength, and temperature. Sorption of heavy metal ions was determined at increasing ratios for Ni^2+^ (41 mg/g), Cd^2+^ (112 mg/g), Cu^2+^ (134 mg/g) and for Pb^2+^ (286 mg/g), with individual adsorption equilibrium equations well approximated by Langmuir isotherms. Adsorption of heavy metals on g-C_3_N_4_ can be promoted or suppressed by a solution’s pH, causing both modified ionic speciation and a change in the surface charge on the adsorbent. Tests showed the adsorption of Pb^2+^ and Cu^2+^ to be relatively low (about 10% of initial mass) at pH 2 and increasing steeply from to ˃90% when the pH increased from 2 to 5, decreasing then slightly at pH ˃ 6 [26].

To avoid heavy metal environmental dispersion, sorption is considered an efficient method for in-situ immobilization of solute metal ions or radionuclides by a reduction from high to low valence states that would cause precipitation and subsequent immobilization on solid particles. Nano zero-valent iron (nZVI) g-C_3_N_4_ composites showed promising pollutant immobilization capacity due to the reduction ability of the former combined with the high sorption capacity of the latter [24].

Carbon-doped g-C_3_N_4_ (C-C_3_N_4_-20) shows an enlarged π-conjugation system that favors aromatic compound adsorption, and a larger specific surface area, which is also an important factor in adsorption performance [27]. Adsorption capacity of C-doped graphitic carbon nitride for MB was compared to that of other adsorbents commonly used in water treatment applications. C–C_3_N_4_-20 showed the highest adsorption capacity (57.87 mg MB/g) among all examined materials: activated carbon, perhaps the most common adsorbent material in water and wastewater processing, had an adsorption capacity of a mere 9.81 mg MB/g. Natural zeolites, another common adsorbent material, showed an adsorption capacity of 29.18 mg MB/g; chitosan-modified zeolite 37.04 mg MB/g. C–C_3_N_4_-20 also showed a significant stability and reusability, maintaining over 93% of its adsorption capacity after 5 cycles of adsorption, water-rinsing and drying [27].

Adsorption of tetracycline (TC), an antibiotic drug widely used not just in human care but also in livestock farming and aquaculture, was observed at levels of 417 mg/g g-C_3_N_4_ at pH 6; adsorption increased by 70% (711 mg/g) when using an KMnO_4_ oxidized composite [28].

Adsorption can be combined with magnetic separation exploiting the combination of the graphitic catalyst affinity towards some compounds and magnetic properties of some metal oxides: g-C_3_N_4_/Fe_3_O_4_ nanocomposites were applied as sorbents for subsequent solid phase magnetic extraction of polycyclic aromatic hydrocarbons (PAHs) from water samples with up to 99.8% PAH removal efficiency [29].

Since photogenerated hydroxyl radicals’ reactions are nonselective, they will virtually react with almost all present organic compounds by H-atom abstraction, direct electron transfer, or insertion, and can promote degradation or complete mineralization of most organic compounds. g-C_3_N_4_ catalysts’ doping often improved the degradation of these contaminants. Photocatalysis is generally more effective when preceded by the adsorption of the target molecule on the catalysts’ surface.

Dyes are often used as test contaminants to assess the photocatalytic properties of a material under visible light, despite some indications that they may not be totally appropriate as model compounds to fully evaluate the activity of novel materials [30]. Notwithstanding the possible influence of a secondary mechanism, i.e., sensitization, that may affect test results depending on dye type, a test model system consisting of a dye and a semiconductor can still be significant to compare the efficiency of a material under different operating conditions, and to assess a catalyst’s ability to induce the degradation of different types of contaminants. Hence, regardless of these limitations, dye tests are still popular, mainly due to the simplicity of their determination methods, and the ease of their visual evaluation.

Consequently, photocatalytic properties of g-C_3_N_4_-based materials have been assessed by several studies involving dye degradation. Jiang et al. determined that photocatalytic degradation of methylene blue by a g-C_3_N_4_–CdS composite was more favorable than that of methyl orange, due to the better adsorption properties of the catalyst for MB, thus confirming the role of adsorption in the overall process [31]. Photodegradation rates have been reported to increase with increasing dye content, as seen at MB concentrations ranging from 10 to 200 mg/L, in the presence of 0.4 g/L g-C_3_N_4_–CdS using 500 W Xenon lamp irradiation. The effect of catalyst dosage, however, is not linear: test results showed that g-C_3_N_4_ increased from 0.1 to 0.4 g/L improved photodegradation efficiency from 70.5% to 90.45% after 180 min exposure, but a further increase of catalyst dosage decreased it again, possibly due to a reduction of light transmission in the suspension. Catalyst doping may improve photodegradation activity: Cu-doped g-C_3_N_4_ was shown to enhance MB degradation under visible light irradiation, achieving close to 100% effectiveness, compared to pristine g-C_3_N_4_ that achieved only 42% removal [32].

The essential contribution of catalysts in photodegradation was demonstrated by Xin and Meng under 300 W Xenon lamp irradiation: photodegradation efficiency of methylene blue (50 mg/L solution in distilled water) was tested with g-C_3_N_4_ synthesized at 520 °C from pyrolysis of cyanamide, dicyandiamide, and melamine, at 1 g/L against a blank (no catalyzer) sample: results showed 25% photodegradation of MB after 5 h irradiation in blank sample (no catalyzer), against 55%, 66%, 60% for the samples dosed with g-C_3_N_4_ derived from cyanamide, dicyandiamide, and melamine, respectively. None of the tested catalysts indicated obvious deactivation during the reaction, suggesting good photochemical stability [33].

Improved g-C_3_N_4_-based photocatalysts for various applications have been developed through suitable structural modifications: novel nanostructured g-C_3_N_4_-based photocatalysts include nanorods (1-D), nanosheets (2-D) and 3-D structures, all created to improve solar radiation absorption, efficient separation of charge carriers, enhance a specific surface area and exposed reactive sites [34]. Considerable progress has been made in this direction in recent years, and the exponential growth of g-C_3_N_4_ composite application is most likely bound to accelerate in future years. An in-depth review and discussion of g-C_3_N_4_ materials synthesis was published by Wen et al. [35]

Various fabrication techniques, e.g., electrospinning, may be used to prepare specific form factor composites, e.g., nanofibers and nanosheets. 1-D nanofibers containing g-C_3_N_4_ and TiO_2_ at variable ratios from a Poly-(vinyl pyrrolidone) (PVP) polymer were produced, showing the enhanced performance of MB removal as a model test for organic pollutant photodegradation [36]. In that study, it was observed that formation of •OH radicals by irradiation under simulated solar light (2.2 kW Xenon lamp at 500 W/m^2^) was maximized when a composite containing 5% g-C_3_N_4_ was used, up to 37.5% more than when TiO_2_-only containing nanofiber was tested. Under those conditions, MB degradation after 20 min irradiation time increased by approximately 200%.

A g-C_3_N_4_/CNT/BiVO_4_ heterostructure photocatalyst containing g-C_3_N_4_, 20 wt% of BiVO_4_ and pre-manufactured carbon nanotubes was synthesized to evaluated phenol removal from solution under simulated solar light irradiation. The addition of CNT in the heterostructured photocatalyst greatly improved the overall specific surface area and inhibited the fast recombination rate of electron-hole pairs. The generated •OH radicals improved degradation of the adsorbed phenolic compound, resulting in the formation of hydroxylated and phenoxy intermediate by-products [37].

Synthesized g-C_3_N_4_ nanosheets containing AgBr were tested for photocatalytic degradation of Methyl Orange (MO) under 100 W tungsten lamp irradiation in different water matrixes. At an initial 7 mg/L MO concentration and 0.7 mg/L catalyst dosage, degradation ranging from 67% in distilled water to 87% in river water was observed after 30 min irradiation at pH 6.0. This apparently surprising result was attributed to the presence of inorganic species in natural water that may have promoted a more intensive production of radical species. The same catalyst composite was also evaluated towards degradation of pharmaceutical compounds such as: metrodinazole, sulfasalazine, cefixime, phenazopyridine, under the same process conditions. The observed degradation showed the following outcomes: Phenazopyridine (79%), Sulfasalazine (59%), Cefixime (34%), Metronidazole (8%), following the trend of estimated compounds oxidation potential, determined as 0.359 V, 0.98 V, −0.56 V and −0.65 V, respectively [38].

Photocatalytic degradation by g-C_3_N_4_ composites using Rhodamine B as a test compound was studied by Xiao et al. [39], comparing several catalysts’ properties, including thermal synthesis environmental conditions (N_2_ or air atmosphere), precursors (dicyandiamide, urea, and guanidine hydrochloride), and hybridized materials supports (MgO, SiO_2_, TiO_2_, Al_2_O_3_). The results showed that TiO_2_ supported g-C_3_N_4_, using dicyandiamide as precursor (dicyandiamide to TiO_2_ mass ratio = 0.75:1, treated in N_2_ atmosphere), has the best reaction activity, due to synergistic effects between the two components, achieving over 95% reduction after 120 min irradiation under Xenon lamp, preceded by 30 min dark adsorption [39].

Photodegradative AOPs are often proposed as suitable processes to remove emerging contaminants, such as endocrine disruptors, pharmaceuticals, pesticides, etc., that cannot be effectively removed by conventional degradation systems [40]. Antibiotic compounds are of particular concern in aqueous media, due to their capability of stimulating bacterial resistance and ecotoxicity, even at low concentrations, and often cannot be degraded without suitable photocatalyst addition [41]. As an example, the antibiotic tetracycline hydrochloride (TC-HCl) is a very stable molecule refractory to common degradation processes. Its degradation was evaluated under visible (λ > 420 nm) simulated solar light irradiation using a 250 W Xenon lamp, with and without a 420 nm UV-cut filter, with the aid of composite Nb_2_O_5_/g-C_3_N_4_ catalysts [42]. This composite’s specific surface area was determined to be 17.184 m^2^/g, about twice that of pristine g-C_3_N_4_, resulting in significantly higher photocatalytic reaction rates. Bare g-C_3_N_4_ resulted in 39.7% TC-HCl degradation after 60 min irradiation, while composites at increasing NbO/CN ratios (1, 3 and 5%) yielded degradations of 67.9, 76.2, and 73.7%, respectively, under the same process conditions.

Ofloxacin (OFL, a quinolone antibiotic) solutions of 10 mg/L in natural river and tap water were tested for degradation under simulated solar light irradiation (1500 W Xenon lamp in Solar Box 1500e, CO.FO.ME.GRA, Milan, Italy), with 0.5 g/L addition of g-C_3_N_4_ synthesized from polymerization of dicyandiamide. Samples were either dark-stirred or sonicated prior to irradiation exposure. An adsorption equilibrium was reached after 20 min stirring or 10 min sonication, at 23 and 11%, respectively. A comparison with results obtained with TiO_2_ catalysts, among the most common in AOPs, was also made. Over 99% OFL removal was observed under 20 min of dark sonication followed by 10 min 500 W/m^2^ irradiation, and the same removal yield was achieved after 10 min dark sonication and 15 min irradiation, or 20 min dark stirring and 20 min irradiation. Without a catalyst, it took approximately 60 minutes’ irradiation to obtain 95% removal. Tests with the TiO_2_ catalyst showed no significant difference between the kinetic constants observed in the two cases, indicating that g-C_3_N_4_ is as efficient a photocatalyst as the former [12].

Melamine-synthesized g-C_3_N_4_ in a solution irradiated with 35 W Xenon lamp source showed the different degradation activity of four pharmaceutical compounds, namely: tetracycline, ibuprofen and salicylic acid in 20 mg/L solutions, and ciprofloxacin in a 10 mg/L solution. These compounds were dosed in 1 g/L g-C_3_N_4_ preparations at pH 5.5, and were left under dark adsorption for 60 min followed by irradiation for up to 4 h. Photodegradation of each pollutant was quite different: ibuprofen showed a minimal decomposition (only 20% after 4 h), followed by salicylic acid (30% decomposition), while ciprofloxacin and tetracycline showed a degradation of 60% and 86%, respectively [43]. Similarly, comparative photodegradation of paracetamol, ibuprofen, and diclofenac was tested with bulk and exfoliated g-C_3_N_4_ and two commercially available TiO_2_ nanomaterials (P25 and CG300) under UV (368 nm, 0.96 mW/cm^2^), and VIS (446 nm, 8.5 mW/cm^2^) irradiation, respectively. 0.9 g/L of each catalyst were suspended in paracetamol (25 g/L), ibuprofen (15 g/L), and diclofenac (25 g/L) solutions subjected to 1-hr dark mixing and up to 6 h irradiation time. Isotherms analysis of the TiO_2_ nanomaterials showed that CG300 absorbed more water (about 23%, a value similar to that of exfoliated g-C_3_N_4_) than P25 (about 5%). Bulk g-C_3_N_4_ showed the least adsorption (at most 2.5%). Adsorption of diclofenac was higher with CG300 (approximately 15 mg/L) than with P25 (approx. 3 mg/L) and g-C_3_N_4_ (2 mg/L), paracetamol was not adsorbed by TiO_2_ catalysts and poorly adsorbed (3 mg/L) by carbon nitride. Ibuprofen was not adsorbed by g-C_3_N_4_ and poorly so by TiO_2_ catalysts (about 2.5 mg/L, each). Paracetamol and ibuprofen were totally removed after 6 h of irradiation, but diclofenac intermediates were observed even after 12 h of continuous exposure. During irradiation, degradation efficiency of g-C_3_N_4_ could be attributed for about 77% to visible light irradiation, and for about 7% to UV effects, in agreement with the respective energy band gaps [44]. Liu and Tang showed up to 98.6% photodecomposition of ibuprofen with g-C_3_N_4_/Bi_2_WO_6_/rGO heterostructured composites at 0.2–2 g/L dosages in river water matrix under solar light irradiation (300 W Xe lamp) after 6 h dark mixing followed by 4 h of irradiation [45].

Tetracycline was degraded by a poly-N-isopropylacrylamide (PNIPAM), magnetic g-C_3_N_4_ composite (PNIPAM/Fe_3_O_4_/g-C_3_N_4_) under visible light irradiation (300 W Xenon lamp). 20 mg/L TC solution with 1 g/L photocatalyst was ultrasonicated for 5 min, and was stirred magnetically in the dark for 1 h and irradiated, achieving a TC mineralization efficiency of approximately 77% [46].

Other studies at the lab scale report that g-C_3_N_4_ is a suitable catalyst building block for treating several other contaminants in aqueous solutions, including VOCs [47,48,49] and Bisphenol-A [50]. Cu-doped g-C_3_N_4_ showed excellent activity for hydroxylation of benzene with H_2_O_2_ as an oxidant at 80 °C, resulting in 74.1% conversion of benzene to phenol with 97.5% yield and for the oxidation of volatile organic compounds (VOCs), such as toluene, styrene and cyclohexane [51].

## 3. Engineered Photocatalytic Process Tests with an Artificial Light Source

Despite the abundance of scientific literature on photocatalysis, its practical use for the degradation of organic pollutants in engineered processes remains a significant challenge due to the current limited capacity for natural light harvesting, and to the limited conversion efficiency of artificial irradiation to photon energy that are necessary to achieve chemical transformations. Despite encouraging laboratory studies, the biggest issues with practical applications of photocatalysis lie with its scaling up to cost- and time-effective industrial processes. The selection of the proper light source is, in addition to the catalyst’s properties, a crucial factor in achieving efficient photocatalysis, since energy efficiency is a big issue for process implementation at an industrial scale. While solar light photocatalysis—a popular research area—could be very energy-efficient using natural rather than artificial irradiation, even in ideal conditions it would require significantly high exposure areas to capture the necessary amounts of energy. For example, it was estimated that to replace one single low pressure UV mercury lamp rated at 1 kW, assuming 40% efficiency, 2.6 m^2^ of exposure area to solar radiation would be needed [52]. This, without considering inevitable process downtime due to nighttime absence or reduction (i.e., during adverse weather) of natural irradiation.

However, even with artificial light sources, UV photocatalytic water treatment is considered impractical at the moment, since other technologies exhibit significantly higher energy efficiency: for example, it was estimated that AOPs involving ozone, chlorine, and/or hydrogen peroxide would typically have electrical energy per order (EEO) values, i.e., the energy required to degrade a contaminant by one log in 1 m^3^ of solution, of 1 kWh/m^3^ or less, while UV photocatalysis may show EEOs in excess of 100 kWh/m^3^ [53]_._ Theoretically, visible light is preferable to UVs for photo-excitation, because higher electricity efficiency and a lower cost can be achieved at longer wavelengths [54]. The band gap of g-C_3_N_4_, unlike the one of TiO_2_, corresponds to a significant width of solar spectrum wavelength, and could thus be used for photocatalytic applications under natural irradiation; however, achieving an adequate and consistent natural irradiation for driving pollutant removal processes can be difficult, and various alternative sources of simulated solar light are often used in photodecomposition studies. These include halogen, Xenon and LED lamps.

Halogen lamps are a class of incandescent sources which use halogen gas to increase light output and rated bulb life. They produce a continuous spectrum of light, from near ultraviolet to deep infrared (approximately 350–1000 nm), with a spectrum similar to that of sunlight. Compared to traditional (incandescent) lamps, they show a higher rated life, a moderately higher efficiency (about 16–38 lumen/W, i.e., 10–20% more than incandescent lamps) and a better light quality. The operating temperature required by the halogen gas reaction is higher than that of typical incandescent bulbs, and hence the heat generated by these lamps is a potential energetic downside to their application, since much of the used up energy is given off as heat. Halogen sources have been tested and used in experimental photocatalytic applications with good results using different catalysts [55,56].

**Figure 1:**
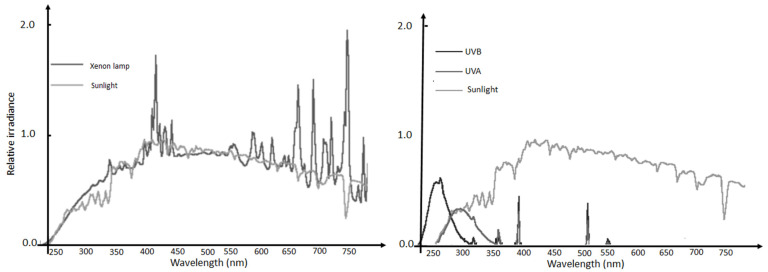
Sunlight spectrum compared to a typical Xenon lamp (**Left**), UVA and UVB sources (**Right**).

Xenon lamps simulate UV and visible solar radiation more closely than other artificial light sources, including halogen lamps (Figure 1), and as such are used in the construction of solar light simulation equipment for industrial testing purposes of materials’ radiation resistance. Xenon lamps do not produce as much heat as halogen sources, and are therefore more energetically efficient, yielding up to 120 lumens/watt source efficiency, hence using less energy to achieve the same irradiation output, and have a rated life of about five fold that of halogen lamps. They also emit minimal UV radiation, which is not needed to activate g-C_3_N_4_; therefore, most published studies on g-C_3_N_4_ photocatalysis refer to the use of Xe-lamps as an alternative to direct sunlight.

Lately, light-emitting diode (LED) sources have evolved into a promising alternative to traditional ones, and have also been used as solar light simulators since they can well reproduce the natural spectrum [17]. LEDs have a much longer lifespan compared to any other lighting technology: last generation LEDs can last 100,000 h, or more, which is 4 to 10 times more than a Xenon bulb. Visible LEDs are extremely energy efficient due to the fact that they waste very little energy in the form of heat and infrared radiation. Typical source efficiency ranges between 37 and 120 lumens/watts, and is subject to far fewer losses from light redirection or reflection than Xenon sources, since LEDs emit light over 180 degrees only, and their compact nature allows the placement of the light sources with a direct focus on the photolytic solution. In addition, LED technology enables the customization of light sources with designed emission peaks at specific wavelengths [57], a possibility that is particularly useful since it provides the option of exploiting specific photocatalytic functions in the process. Due to their lower light-scattering effects, LEDs also represent a suitable candidate for effective processing of water matrices with residual turbidity [54].

Although LEDs’ effectiveness has been confirmed in water disinfection [58], specific LEDs utilization in water treatment is still under development: most studies so far have been conducted with UV-LEDs, in combination with a TiO_2_ catalyst or added oxidants (e.g., H_2_O_2_, PS, PMS) [59]. There is very little experience on the applicability of Vis-LED photocatalysis, although it was reported that carbon quantum dots (CQDs), a novel carbon nanomaterial with improved light harvesting capabilities, could boost the photocatalytic performance of g-C_3_N_4_ dramatically, enabling CQDs/g-C_3_N_4_ composites to work highly effectively in the degradation of refractory pollutants in water under such sources [60]. Activation of peroxymonosulfate (PMS) by g-C_3_N_4_ under 400 nm LED irradiation was tested as a sulfate radical anion AOP for the degradation of organic pollutants (Acid Orange 7) [61]. In this respect, further studies on pollutant removal and EEO estimations of the process will be required [62].

It should be noted that radiation is emitted at different wavelengths by each specific irradiation source; for this reason, a comparison of experimental results reported by different studies is not always immediate nor significant, as often only basic characteristics of light sources (e.g., lamp type and power rating) are reported, while actual emission spectra are not.

### 3.1. Laboratory Tests of Dye Degradation under Xenon Lamp Light Irradiation

**Figure 2:**
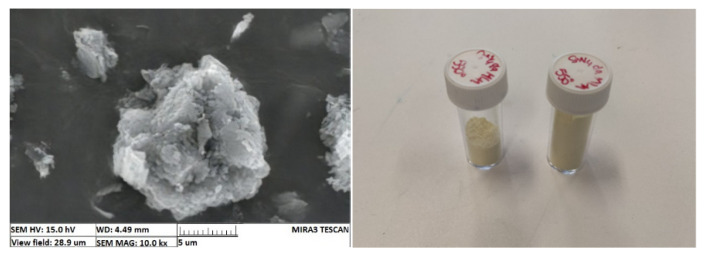
Vials of synthesized g-C_3_N_4_ (**Left**) and SEM image of the flocs (**Right**).

In order to assess firsthand the factors influencing contaminant removal in a g-C_3_N_4_-based photocatalyst process, batch tests were carried out exposing a methylene blue solution to with lab-synthesized g-C_3_N_4_ to a Xenon light source. Dicyandiamide-derived g-C_3_N_4_ was produced and characterized according to the process described by Sturini et al., which included X-ray diffraction, FT-IR and UV-visible diffuse reflection spectrum analyses [12]. The resulting material is shown in Figure 2 Left. This material was then exfoliated mechanically to reduce the particles’ size to a few microns in order to increase its specific surface, yielding microparticles, which would facilitate its subsequent recovery by filtration, compared to the nanoscale materials commonly used nowadays (Figure 2 Right).

Methylene blue was selected as a test pollutant to assess the effectiveness of the produced carbon nitride and of the irradiation source. Initial tests carried out with an UVC (15 W) irradiation source (100–280 nm) of pure MB solution (10 mg/L) and of 0.5 mg/L g-C_3_N_4_ added solution showed no difference between homogeneous and heterogeneous photocatalysis process conditions, yielding a very similar adsorbance reduction (−58 and −64%, respectively) after 60 min, the difference being mainly due to adsorption of the pollutant on the catalyst. These results confirm the trends reported by other researchers under similar conditions [63]. This result confirmed that a more suitable light source in the visible spectrum was needed to induce g-C_3_N_4_ photocatalysis. A 150 W, 3800 °K commercial Xenon light source (Ledlux, Milan, Italy) was therefore adopted.

**Figure 3:**
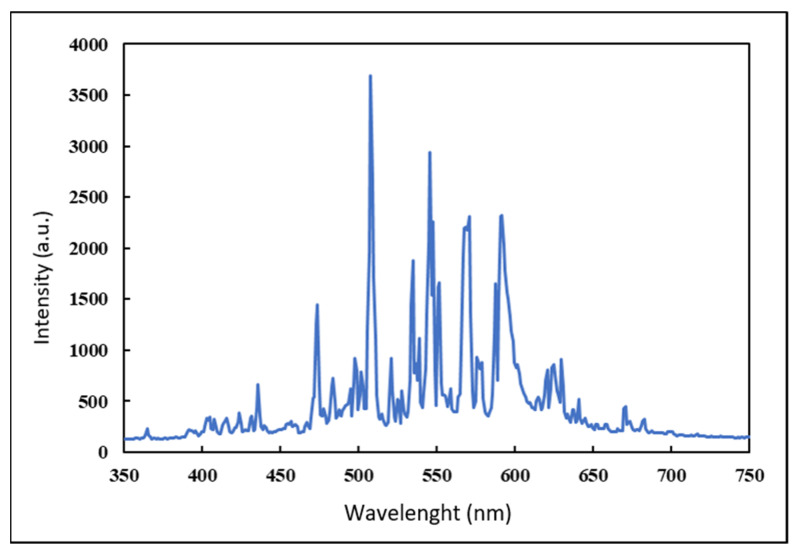
Measured emission spectrum of the 150 W Xenon bulb used in tests.

Heterogeneous photocatalysis batch tests were then conducted in stirred beakers containing MB solutions, at an initial temperature of 20 °C. Magnetic stirrers also allowed reoxygenation of the solution, since molecular oxygen acts as a scavenger, limiting the recombination of electrons, thus increasing the efficacy of the process. The light source was placed above the beakers (10 cm over the upper rim), and the entire setup was contained within a dark box to avoid the influence of external light. The Xenon bulb has a higher emission (14,000 lumen) compared to a common commercial halogen lamp with the same power rating, and its visible spectrum approximates well that of natural solar light, as shown in Figure 3, reporting the actual observed spectrum of the light source as detected by a high resolution spectrometer (350–1000 nm).

Tests were conducted under different g-C_3_N_4_ concentrations and at different pH values. In view of the fact that visible light irradiation may also induce some MB degradation, a control photolysis-only test (Xenon irradiation alone, no catalyst addition) was conducted on the MB solution to highlight the contribution of g-C_3_N_4_ to the removal and degradation of the dye.

**Figure 4:**
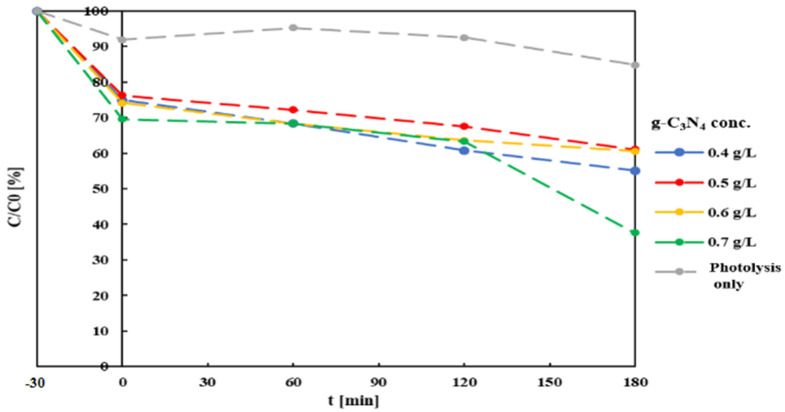
Residual MB concentration after Xe-light photocatalysis at different g-C_3_N_4_ concentrations and initial pH = 6.8. Time 0 indicates the start of the irradiation period, while dark mixing period started at t = -30 min.

Four 200 mL samples were prepared from a MB solution (10 mg/L) in distilled water, adding different amounts of g-C_3_N_4_ to reach final catalyst concentrations of 0.4, 0.5, 0.6 and 0.7 mg/L. Each sample, at initial pH = 6.8, was subject to 30 min intense mixing under dark conditions, to allow the establishment of an adsorption equilibrium. Sample aliquots were collected at the end of this phase, and subsequently after 60, 120 and 180 minutes’ irradiation, and were then filtered with a 0.22 µm PTFE syringe filter to eliminate suspended particles. Residual MB concentration was measured by spectrophotometer (UVmini-1240, Shimadzu Europe), which had been previously specifically calibrated. This, and all the following procedures, were conducted in triplicate. A final balance on sample volumes indicated that a volume reduction of about 5% resulted from water evaporated due to the heat emitted from the light source. Figure 4 summarizes the results of the first set of test (variable catalyst concentration) compared to the catalyst-free sample. Concentration values were calculated directly from adsorbance values.

**Figure 5:**
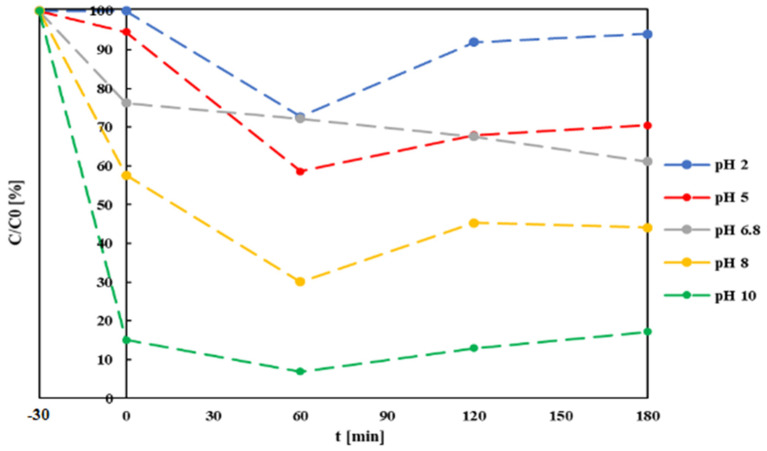
MB residual concentrations after adsorption and photocatalysis at different initial pH values and g-C_3_N_4_ concentrations of 0.5 mg/L.

A second batch of tests was conducted on 4 samples of the same MB solution with 5 mg/L g-C_3_N_4_ addition. The same procedure described above was carried out with pH adjusted at 2, 5, 8 and 10. Substantial differences in process development could be observed at increasing pH values, concerning both the adsorption and photocatalysis components in the second set of tests (Figure 5). At the increasing pH value, the adsorption increased significantly, from approximately nil at pH = 2.0 to 83% at pH = 10. The rate of increase is more than proportional to the pH value, indicating a significant influence of the latter on adsorption mechanisms, confirming the trend effects previously reported by Soltani and Entezari [64]. An increase in adsorption capacity with increasingly alkaline pH values could be attributed to the strong electrostatic attraction of negatively charged MB molecules and positively charged g-C_3_N_4_ particles; such electrostatic phenomena could also influence the production of radical species in the solution, improving reaction kinetics [65]. The optimal pH for the overall photocatalytic reaction, however, will depend on the specific target molecule to be removed: a study on g-C_3_N_4_-assisted photocatalytic reduction of ibuprofen showed that acidification of the solution had the opposite effect, improving dark adsorption until reaching an optimal pH value of 2.5 [66].

Under simple photolysis, MB removal was low (14% after 180 min). This result is in line with previous studies reporting just 10% decoloration of an MB solution after 150 min of exposure to direct sunlight [63]. Wang et al. reported 20% decoloration of an MB solution after 180 min of irradiation with a 350 W Xenon lamp [67]. Comparison of full photocatalysis tests with the former shows a clear contribution of g-C_3_N_4_ to the process: first through the adsorption mechanism (after the first 30 min of dark mixing, MB concentration already shows 25–30% reduction), afterwards by actual degradation, which closely approximates first-order reaction kinetics. Degradation rate values increase with the decreasing catalyst concentrations. These results can be compared with those reported by Paul et al., which observed an increase in adsorption kinetics with the increasing (from 0.1 to 0.5 mg/L) catalyst concentration, diminishing when further increasing it to 0.7 mg/L [68].

Significant solution decoloration was observed in the first 60 min of irradiation, representing photocatalytic reaction effects, which were more pronounced at lower pH values. This could be attributed to more efficient photocatalysis under these pH conditions, but also to progressive saturation of the catalyzers’ active sites at a higher pH. At 120 and 180 min irradiation times, MB concentrations in solution increased, possibly due to desorption of part of the molecules previously fixed onto the catalyst’s surface, due to mixing turbulence. This phenomenon is more pronounced at acidic pH values and is not visible at unmodified solution pH, possibly indicating that a pH around neutrality (6.8 in the test) represents optimal conditions for carrying out photocatalysis of the dye.

A possible photodegradation mechanism of MB was discussed by Xin and Meng [33]: graphitic carbon nitride, with a band gap around 2.7–2.8 eV, shows absorption properties in the blue region of the visible spectrum. Photoexcitation leads to charge separation between the electron in the conduction band and the hole in the valence band, suggesting that N atoms would be preferential oxidation sites, and C atoms, reduction sites. Active species, such as hydroxyl radicals (•OH), superoxides (O_2_• or HOO•), and holes, are formed during photodegradation. O_2_• is initially produced from dissolved O_2_ and a photoinduced electron. In a second step, H^+^ transfers a second electron to form H_2_O_2_, which is subsequently activated to •OH by accepting a third electron, a step that g-C_3_N_4_ is capable of inducing, due to its band positions. The photodegradation mechanism of MB can therefore be explained by a photogenerated multistep reduction of O_2_: the •OH radical is nonselective, and will react with almost all organic compounds, in this case promoting the mineralization of MB.

Solution pH varied at different rates during tests, as shown in Table 1. In tests carried out at an acidic pH, its values remain fairly constant throughout. On the contrary, at alkaline pH (8 and 10), progressive acidification occurs during the evolution of the tests, with a maximum drop of 2.1 units after 180 min, in the test with pH_0_ = 10. This phenomenon may be attributed to the production of H^+^ ions resulting from MB degradation, according to the reaction proposed by Galagan and Su [69]:

(1) $$C_{16}H_{18}N_{3}S^{+}+\frac{15}{2}O_{2}\to 16{\mathrm{CO}}_{2}+3{\mathrm{NO}}_{3}{}^{-}+{\mathrm{SO}}_{4}{}^{2-}+6H^{+}+6H_{2}O$$

The tests performed confirmed the validity of Xenon sources to perform photocatalytic processes with g-C_3_N_4_, however, they point out that even though this light source is more efficient than halogen sources, it still causes significant energy dispersion in the form of heat. In turn, heat caused evaporation of the solution, reducing the apparent process efficiency by about 5%, due to the observed water loss. This evidence indicates that the use of LED irradiation sources may lead to more efficient processes, both energetically and in terms of effectiveness. Tests also confirmed that pH control in the solution during the reaction is an important parameter that should be optimized to maximize process efficiency. The pH not only affects photodegradation of the contaminants, but also the evolution of the dark adsorption phase. It can be assumed, based on the existing literature, that pH effects are contaminant-dependent, and hence optimal process conditions ought to be investigated on a case-by-case basis [24,25,26,28]. pH control can be implemented fairly easily in most processes by means of simple instrumentation and PID control strategies [70]. Finally, MB degradation is affected by the amount of catalyst present, increasing with increasing catalyst amount, up to a point. While an increase in catalyst amount increases the number of active sites, causing an increase in the number of •OH radicals which are formed and can take part in the process, beyond a certain limit the solution’s increased turbidity may impair the radiation’s penetration, decreasing the reaction rate.

### 3.2. Considerations for Heterogeneous Photocatalytic Reactors Improvement

Photocatalytic reactors can be classified according to the catalyst’s form in the reactor (suspended or immobilized), and by the irradiation configuration (submerged/external or mixed sources, such as external lamps with optical fibers inside the reactor). Reactors with suspended catalyst particles generally have a good mass transfer (reactant/catalyst contact), but a poor photon transfer (i.e., catalyst irradiation); in addition, they need post treatment (filtration) to separate the photocatalyst’s particles from the liquid. Reactors with an immobilized catalyst generally have lower contact efficiency but better photon transfer, and do not need post treatment. Several materials (i.e., glass beads/disks/walls, cloths, meshes, porous or ceramic structures) can be coated with catalysts for such a purpose. Notwithstanding the low density of g-C_3_N_4_ particles, significant stirring was needed to keep this material in suspension. Good contact between catalyst and solution was achieved, as proven by the high initial adsorption (30–40% at pH = 6.8, up to 90% ay pH = 10), but poor photon transfer was achieved, as observed by the limited effect of subsequent phoyolysis reactions on MB degradation.

Light penetration into the suspension is also of concern. Often, photocatalysis tests are performed in laboratory settings in partially filled, stirred beakers, as in this case. However, typical visible radiation has an effective water penetration depth of about 9 mm, only activating a thin layer close to the light source [71]. Mixing alone may thus not be sufficient to effectively expose the entire volume of the solution to the light source. This also could explain the relatively limited effect of photocatalysis versus initial adsorption shown in our laboratory tests.

The light source itself is an important factor in photocatalytic systems, as it determines for a large part the efficiency and application of the technology. LED sources at the moment appear to express the most potential for the efficient design of photocatalytic reactors, as they combine a unidirectional emission, which is highly compatible with laminar flow reactor devices, low heat emission, and customizable spectral characteristics that could maximize a specific catalyst’s activation.

## 4. Discussion

Notwithstanding the considerable progress made in recent years, there are still many challenges to achieve a thorough understanding of the underlying mechanism of photocatalysis and in particular of g-C_3_N_4_-based photocatalysis. In spite of promising results so far, studies in this field are still in the preliminary stages.

The many studies reported in the literature have been conducted with different solar light simulator sources with rated capacities differing for up to an order of magnitude, and under non-standardized, or non-verified, emission spectra. Studies conducted so far have highlighted the potential of the material towards the degradation of several types of pollutants, but a clear relationship between energy inputs, spectral fingerprint intensity of the light source, radical generation and chemical properties of the target pollutants has not yet emerged. More specific studies are needed to take full advantage of the obvious outstanding structural and electronic properties of this material in composites, and their relationship with the active-site structures and composition.

Although visible light simulation sources seem to be more efficient than artificial UV sources, the lack of energy cost comparisons between photocatalytic technologies is necessary before industrial applications can be planned. The electrical energy per order (EE/O), expressed as kWh, needed to degrade a pollutant’s mass by one log in a unit volume of contaminated solution, could be a proper parameter to evaluate in this respect.

Understanding the influence of solution conditions (in particular, pH) on the relative role of the two stages (adsorption and photolysis) during the photodegradation process ought to be better understood. From tests conducted by the authors, it seems that an alkaline environment would favor the initial adsorption process, while an acidic environment would enhance subsequent photolysis. Dynamic control of this parameter during the photocatalytic process could enhance its overall efficiency.

Photocatalytic processes in the treatment of contaminated solutions can be carried out in two modes: with suspended catalysts particles in the solution or in fixed-bed reactors, where catalysts are immobilized on solid supports. Generally speaking, suspended catalyst reactors are considered more efficient, but they require post-separation of the particles, prior to effluent discharge, which may require specific processes (nanofiltration, in case of nanocatalysts particles, or magnetic separation, as in the case of iron oxides-loaded composites), and therefore the development of fixed-bed catalysts is becoming an interesting area of development, which includes the application of modern additive manufacturing technologies. The supporting materials could also favor catalysts’ stability and long-term reuse. Researchers have postulated the possibility of fabricating membrane photocatalytic reactors, coupling photocatalysis with membrane separation as a possible technology for industrial applications with the development of tailor-made materials [72]. Such materials could find an ideal field of application in advanced water reuse practices for specific end uses [73]. On the other hand, application of low-cost visible-light activated photocatalysts may be efficiently exploited in various situations of water contamination occurring in areas where insolation is abundant and persistent, such as in some low latitude countries. Limited light penetration in the solution suggests that thin laminar flow reactors would be best suited to achieving effective photocatalysis.

## 5. Conclusions

Properties, advantages and applications of g-C_3_N_4_-based photocatalysts were reviewed in this paper, and an application experiment for methylene blue dye degradation was conducted, which confirmed results achieved by previous studies. g-C_3_N_4_ composites have been proven to be among the most promising advanced photocatalytical materials for many applications. The green synthesis capability of these novel compounds with outstanding intrinsic properties, high chemical stability/photocatalytic capacity, and reusability is constantly improving. Synthesis and engineered modification (element-doping and structural) of g-C_3_N_4_, and application of its composites in the efficient elimination of heavy metal ions, radionuclides and persistent organic pollutants from aqueous solutions or soils by visible-light-driven photocatalysis, anticipate huge prospects in sustainable wastewater purification due to the high photocatalytic capacity of this material. However, real-scale environmental uses of this technology are still constrained by technical and practical issues that require further investigation.

Photocatalytic removal of pollutants is a great technical challenge, since acting photocatalytic mechanisms and relationships between heterostructures and photocatalytic degradation capacity are still unclear. Furthermore, the detection of intermediates during photocatalytic processes, and the determination of possible photocatalytic routes for many pollutant removal is necessary to understand the reactions’ mechanism. Possible toxicity of photocatalytic by-products should be considered for those pollutants that cannot be fully mineralized by photodegradation.

The tests performed showed both the effectiveness and the limits of a suspended g-C_3_N_4_ catalyst reactor. In particular, light penetration in the reactor’s volume seems to be a limit to the complete development of photocatalysis after pollutant adsorption onto the catalyst’s surface. The design of thin-layer flow reactors, allowing full penetration of light radiation could improve the efficiency of the process, possibly also reducing the necessary reaction time.

Photoreactor engineering and design, in particular specific irradiation intensity requirements and energy efficient irradiation sources, should be carefully investigated. Specific composite formulations may be required for optimal removal of specific classes of pollutants, and structural form design (1-D, 2-D or 3-D), with the immobilization of high specific superficial area catalysts in the reactor structure, is of great interest and may play an important role in enabling the use (and reuse) of the technology in industrial applications. In particular, durability/reusability of g-C_3_N_4_-based immobile composite materials could be an important factor in decreasing the cost of catalysts in real applications. Catalyst immobilization would also reduce potential toxicity risks to ecosystem and organisms, as photocatalysts in solution may be released into the natural environment if not completely removed prior to discharge.

Initial evidence suggest that visible light/g-C_3_N_4_ processes may soon become a valid alternative to the more studied UV/TiO_2_ AOPs, and there is little doubt that the explosive growth of interest in g-C_3_N_4_-based composite photocatalysts will continue to accelerate in the near future. Research to transfer this technology from the laboratory scale to the chemical and environmental engineering realm for process scale-up is needed.

## Figures and Tables

**Table 1:** Observed pH variation during the second batch of tests.

Elapsed Time (min)	pH			
−30 (t_0_)	2.0	5.0	8.0	10.0
0	2.3	4.5	7.8	10.4
60	2.2	4.8	8.0	9.3
120	1.9	4.6	7.3	7.6
180	2.0	4.8	7.2	7.9

## Data Availability

Data are summarized in Figure 4 and Figure 5. They can be requested by contacting the corresponding author.
